# Optimization Design of Metakaolin-Based Geopolymer Solidification for Potassium Copper Hexacyanoferrate After Cs^+^ Adsorption Using Response Surface Methodology

**DOI:** 10.3390/ma19071469

**Published:** 2026-04-07

**Authors:** Yuqing Liao, Xingyu Yu, Xinyi Yuan, Jingsong Wang, Yao Yan, Gaoshang Ouyang

**Affiliations:** 1 School of Civil Engineering, University of South China, Hengyang 421001, China; 13575148177@163.com (Y.L.);; 2 School of Resource Environment and Safety Engineering, University of South China, Hengyang 421001, China

**Keywords:** geopolymer, metakaolin, response surface methodology, Cs

## Abstract

**Highlights:**

**What are the main findings?**

- A quadratic regression model based on RSM optimizes the geopolymer mix for Cs immobilization with a high predictive accuracy (R^2^ > 0.99).
- The H_2_O/Na_2_O ratio is the most critical factor affecting compressive strength and leaching resistance, followed by Na_2_O/Al_2_O_3_ and SiO_2_/Al_2_O_3_.
- The optimal mix (Na_2_O/Al_2_O_3_ = 0.84, SiO_2_/Al_2_O_3_ = 2.8, and H_2_O/Na_2_O = 10.23) yields MPa a strength of 23.41 MPa and ultra-low 42-day Cs leaching.

**What are the implications of the main findings?**

- A quantitative mix design methodology for the efficient solidification of Cs-laden adsorbents using metakaolin-based geopolymer is provided.
- Microstructural analyses (SEM/XRD/FT-IR) confirm effective Cs encapsulation, supporting the geopolymer’s stability in nuclear waste treatment.
- The model offers a scalable strategy for immobilizing other radionuclides (e.g., Sr^2+^ and Co^2+^) in multi-nuclide radioactive wastewater.

**Abstract:**

This study employed a metakaolin-based geopolymer (GP) to solidify potassium copper hexacyanoferrate after its saturation with adsorbed Cs^+^. The experiment was designed using response surface methodology (RSM) in the Design–Expert 13 software, targeting the compressive strength and cumulative leaching fraction of the solidified form. A regression model was developed to achieve the multi-objective optimization of the comprehensive performance of the GP solidified product. Regression analysis identified the optimal mix proportion as Na_2_O/Al_2_O_3_ = 0.84, SiO_2_/Al_2_O_3_ = 2.8, and H_2_O/Na_2_O = 10.23. Under these conditions, the experimentally measured compressive strength was 23.41 MPa. The 42-day cumulative leaching fractions at 25 °C and 40 °C were 7.906 × 10^−4^ cm and 1.5923 × 10^−3^ cm, respectively, both significantly below the national standard threshold (Standard Code GB7023-2011) of 2.6 × 10^−1^ cm. The percentage error remained within 10%, indicating strong agreement with predicted values. These results suggest that metakaolin-based GP exhibits promising potential for the immobilization of radionuclides.

## 1. Introduction

Nuclear energy, as a significant alternative energy source, is receiving increasing global attention. However, the nuclear fuel cycle generates a considerable amount of radioactive solid waste and wastewater [1,2]. Among the radionuclides produced, ^137^Cs is a major fission product. It has a long half-life (approximately 30 years), is highly soluble in water, and accumulates in biological systems. The β and γ radiation emitted by isotopes, such as Cs, can penetrate cells, causing DNA strand breaks and altered gene expression, and potentially leading to cell death or carcinogenesis. Furthermore, Cs contamination resulting from nuclear accidents (e.g., Fukushima, Japan) may spread extensively via atmospheric deposition, contributing to combined pollution in ecosystems, such as forests and wetlands [3,4]. Therefore, the effective treatment of Cs in radioactive wastewater is of critical importance for both public health and environmental safety.

Adsorption is a widely applicable and relatively simple method for treating radioactive wastewater, offering efficient volume reduction in radioactive waste [5,6]. Prussian blue (PB), a metal hexacyanoferrate compound (Fe_4_[Fe(CN)_6_]_3_) originally used as a dye, demonstrates high selectivity for Cs^+^ due to the favorable match between the cubic lattice of its structure and the hydrated radius of Cs^+^ ions [7]. Among PB analogs, potassium copper hexacyanoferrate (KCuFC) exhibits a superior adsorption capacity and mechanical stability compared to other hexacyanoferrates. Its robust hexacyanoferrate framework supports both high adsorption efficiency and material durability, making it a promising candidate for the long-term remediation of radioactive wastewater [8,9]. The final disposal of spent adsorbents typically involves immobilization within a solid matrix followed by deep geological disposal [10,11]. Thus, the incorporation of effective inorganic adsorbents into stable solidified matrices enhances long-term containment.

Geopolymers (GPs) are an emerging class of amorphous inorganic aluminosilicate polymers characterized by a tetrahedral network of AlO_4_ and SiO_4_ units. They are typically synthesized through the reaction of aluminosilicate precursors with alkali activators, forming either a zeolite-like three-dimensional network [12,13] or a semi-crystalline structure [14]. GPs are known for their high compressive strength [15], thermal resistance [16], chemical durability [17], and low permeability [18]. These advantageous properties have resulted in the accelerated development of GPs as effective solidification matrices for hazardous waste, drawing increasing interest from researchers around the world. Previous studies have confirmed the superior immobilization and stabilization performance of GPs for various radionuclides and heavy metals, including Cs, Sr, Pb, Cd, Co, and Cr [19]. For example, Shao et al. [5] demonstrated that metakaolin/slag-based GP microspheres (GPMs) can effectively adsorb Sr^2+^ and Cs^+^. The GPMs can be subsequently solidified within a GP matrix, resolving compatibility issues between the adsorbent and matrix, and enhancing slurry workability. Leaching tests further confirmed the strong immobilization capacity of the slag-based system. Similarly, Dang et al. [20] synthesized a novel cesium adsorbent (AMP-HMSS) and immobilized the spent AMP-HMSS/4-Cs within a fly ash-based GP, which showed excellent Cs^+^ immobilization performance. However, most current studies do not thoroughly examine the relationship between mix design parameters and the synergistic solidification behavior of radionuclides. As such, the development of a unified theoretical framework for optimal GP mix design remains ongoing.

Response surface methodology (RSM) is a statistical method for experimental design, modeling, and optimization. After identifying key variables and their levels, RSM enables the design of experiments that yield regression models and response surfaces, allowing researchers to elucidate the functional relationships between process parameters and response values. These models, based on polynomial regression, can then be used to optimize response outcomes [14,21]. RSM is particularly effective for multi-factor, multi-level experiments with continuous response variables, offering significant advantages over traditional one-factor-at-a-time or orthogonal design methods. In recent years, RSM has been increasingly applied to GP mix design [22,23,24]. Existing research indicates that molar ratios, such as Na_2_O/SiO_2_, SiO_2_/Al_2_O_3_, and H_2_O/Na_2_O, along with their interactions, significantly influence both the mechanical strength and leaching resistance of metakaolin-based GPs. Specifically, Na_2_O/SiO_2_ governs the reactivity of alkali activation [25], SiO_2_/Al_2_O_3_ determines the density of the three-dimensional framework [26], and H_2_O/Na_2_O affects the reaction kinetics and pore structure [27]. Optimizing these ratios synergistically enhances compressive strength and minimizes radionuclide leaching. By applying RSM to design the mix composition of a GP solidification matrix for Cs^+^-saturated KCuFC, a quantitative relationship can be established between formulation parameters, mechanical performance, and leaching behavior. Moreover, investigating the structure–activity relationship between the GP and the adsorbent can clarify the influence mechanisms of individual and interactive factors on the composite material’s properties.

In this study, RSM was employed to design and analyze experiments, using a polynomial regression model to evaluate the effects of Na_2_O/SiO_2_ (X), SiO_2_/Al_2_O_3_ (Y), and H_2_O/Na_2_O (Z), along with their interactions, on the mechanical and leaching properties of metakaolin-based GP. Based on model’s validation and optimization, an optimal mix ratio was determined. Experiments were conducted using this optimized formulation, and the GP’s microstructure and Cs immobilization mechanism were analyzed using techniques such as X-ray diffraction (XRD) and scanning electron microscopy (SEM). The findings provide insight into the potential application of metakaolin-based GPs for immobilizing KCuFC-Cs and offer a theoretical foundation for designing optimized radionuclide immobilization systems, and the long-term evolution of the optimized geopolymer under extended aging conditions, which represents a clear direction for subsequent investigation, will be the focus of future research.

## 2. Models and Methodology

### 2.1. Response Surface Methodology

This study employed the standard experimental design method of RSM, specifically the Box–Behnken Design [28]. This approach establishes a functional model to describe the relationship between variables x_1_, x_2_, …, x_k_ and the response y, allowing for the analysis of both individual and interaction effects among the variables. Three critical factors were selected: Na_2_O/SiO_2_ (A), SiO_2_/Al_2_O_3_ (B), and H_2_O/Na_2_O (C). Each factor was assigned three levels, coded as +1 (high), 0 (center), and −1 (low). The molar ratios of Na_2_O, Al_2_O_3_, and SiO_2_ were designated as independent variables A, B, and C, respectively, as shown in Table 1. The response variables included the 28-day compressive strength (y_1_), and the 42-day cumulative leaching fractions at 25 °C (y_2_) and 40 °C (y_3_). A three-factor, three-level Box–Behnken central composite design was implemented for the experimental scheme, involving 15 runs including three replicates at the center point. The standard deviation (σ) and coefficient of variation (CV) of the response values were calculated based on repeated measurements under identical input conditions to estimate experimental error. The relationships and interactions among Na_2_O/SiO_2_, SiO_2_/Al_2_O_3_, and H_2_O/Na_2_O, and the response variables, were modeled using a quadratic polynomial equation (Equation (1)). The optimization objective was to maximize the compressive strength and minimize the cumulative leaching fraction, enabling the prediction of an optimal GP formulation.

(1) $$y=\beta_{0}+\sum_{i=1}^{k}\beta_{i}x_{i}+\sum_{i=1}^{k}\beta_{ii}x_{i}^{2}+\sum_{j=2}^{k}\sum_{i=1}^{j-1}\beta_{ij}x_{i}x_{j}$$

In this equation, *y* is the predicted response, *β*_0_ is the intercept, *β_i_* represents the linear coefficients, *β_ii_* represents the quadratic coefficients, *β_ij_* represents the interaction coefficients, and *x_i_*, and *x_j_* are the coded independent variables listed in Table 1.

RSM optimization consists of three main steps: (1) statistically designing the experiments, (2) estimating the model coefficients, and (3) predicting the responses within the experimental domain and assessing model adequacy [29]. Experimental runs were conducted according to the design matrix, and response surface analysis was performed using nonlinear regression to derive the fitting equation. The interactions between factors were fitted with a second-order polynomial, and the optimal combination was obtained based on this regression model.

### 2.2. Materials

This study evaluated the Cs^+^ immobilization efficiency of metakaolin-based GP solidified with Cs^+^-adsorbed KCuFC. KCuFC was synthesized using a previously reported method [30]. Saturation adsorption experiments were carried out under the following conditions: temperature = 298 K, solution pH = 7, cesium ion concentration = 1 g/L, and adsorbent dosage = 1.75 g/L. A concentration of 1 g/L Cs^+^ was selected to assess the material’s maximum encapsulation capacity under stress conditions, representing realistic levels in certain high-level wastes, to provide valuable safety margin data for applications involving concentration factors. This process yielded KCuFC-Cs with an adsorption capacity of 143.8 mg/g. This value represents the maximum uptake capacity achieved under the defined experimental parameters and provides a quantitative basis for subsequent GP solidification studies. The chemical reagents used are listed in Table S1.

**Figure 1:**
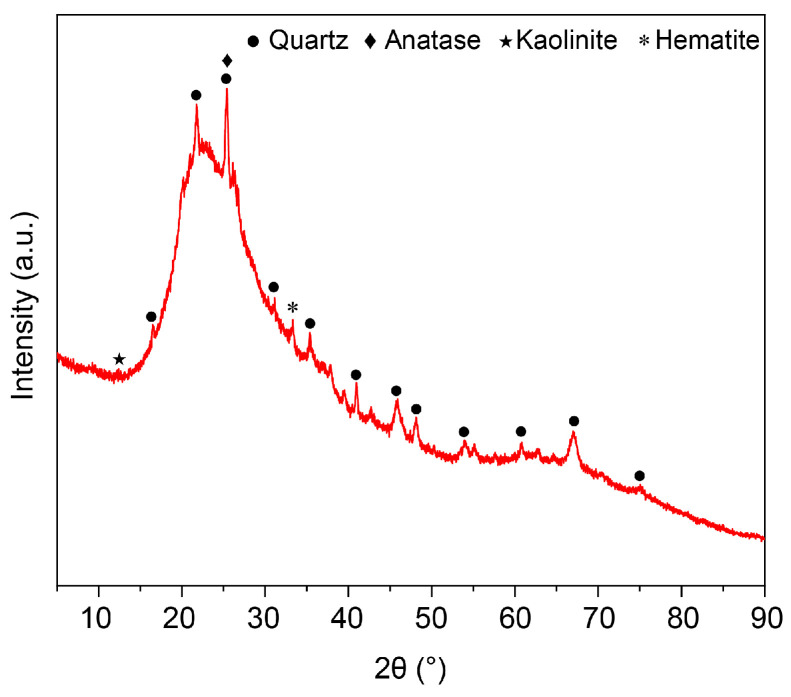
X-ray diffraction pattern of metakaolin.

Metakaolin (MK), supplied by Shanxi Super Brand Calcined Kaolin Co., Ltd. (Linfen, China), was used as the main precursor. Its phase composition is shown in Figure 1, and the chemical compositions of metakaolin and silica powder determined by X-ray fluorescence spectroscopy (XRF) are provided in Table 2. The alkali activator (modulus 1.2) was prepared by mixing sodium hydroxide (NaOH), sodium silicate solution (water glass), and distilled water.

### 2.3. Experimental Process

GP solidified forms were prepared using metakaolin, KCuFC-Cs (12 wt%), and silica powder in varying proportions, with sodium silicate and NaOH as the alkali activators. The preparation process was as follows: the dry components were mixed uniformly in a cement mortar mixer. The resulting slurry was poured into cylindrical molds (20 mm diameter × 20 mm height) and compacted using a vibrating table to remove any entrapped air. After the initial setting, the samples were demolded and cured at 25 °C and 90% relative humidity for 28 days. All the GP samples underwent a systematic surface pretreatment to minimize the influence of surface irregularities and loosely adherent particles. The specimens were carefully polished using 400-grit silicon carbide paper, followed by unidirectional wiping with a nonwoven fabric saturated with absolute ethanol to remove any surface contaminants. Finally, the samples were oven dried at 40 °C until they reached constant weight. Compressive strength testing was conducted in accordance with the Chinese National Standard GB/T 17671-2021 [31]. Cylindrical samples (10 mm radius × 20 mm height) were prepared, and six parallel specimens per mix were tested to determine average compressive strength after 28 days.

The leaching performance was evaluated based on Chinese national standard GB 7023-2011 [32] and Chinese national standard GB 14569.1-2011 [33], which specify the long-term leaching procedures and performance requirements for cement solidified forms. Three replicate samples from each group were immersed in 300 mL of deionized water in polyethylene containers, maintained at 25 °C and 40 °C. The leachant was replaced at cumulative time intervals of 1, 3, 5, 7, 10, 14, 21, 28, 35, and 42 days.

### 2.4. Analytical Methods

The microstructure, morphology, and phase composition of the GP solidified forms were analyzed using multiple techniques. X-ray diffraction (XRD; Shimadzu XRD-6100, Kyoto, Japan) was used to identify crystalline phases in six sample groups: metakaolin, S1, S2, S4, S8, S10, and S12. Scanning electron microscopy (SEM; Thermo Scientific Quattro S, Waltham, MA, USA) provided microstructural images, and energy dispersive X-ray spectroscopy (EDS; Oxford INCA X-MAX, Abingdon, UK) was used to determine elemental composition and distribution.

Fourier transform infrared spectroscopy (FT-IR; Shimadzu IRTracer-100, Kyoto, Japan) was employed to analyze the changes in the molecular structure and functional groups across the same six groups. Pore density was characterized via Brunauer–Emmett–Teller (BET; Micromeritics ASAP2460, Norcross, GA, USA) surface area analysis combined with nitrogen adsorption–desorption isotherms. The concentration of Cs^+^ in the leachate was measured using inductively coupled plasma mass spectrometry (ICP-MS). The leaching kinetics were interpreted based on the semi-dynamic leaching model, with the kinetic expressions given in Equations (2) and (3).

The cumulative leaching fraction CLF (cm) is calculated as follows:

(2) $$CLF=\frac{\sum a_{n}/A_{0}}{S/V}$$

The leaching rate R_n_ (cm/d) is calculated as follows:

(3) $$R_{n}=\frac{a_{n}/A_{0}}{S/V}\times \frac{1}{t_{n}}$$

where a_n_ is the mass of Cs leached during the n-th leaching interval (g), A_0_ is the initial mass of Cs in the solidified waste form (g), S is the contact area between the waste form and the leachant (cm^2^), V is the volume of the waste form (cm^3^), and t_n_ is the cumulative leaching time corresponding to the n-th interval (days).

## 3. Results and Discussion

### 3.1. Effects of Single Factors on Cesium Leaching

#### 3.1.1. Effect of Na_2_O/Al_2_O_3_ on Cesium Leaching

**Figure 2:**
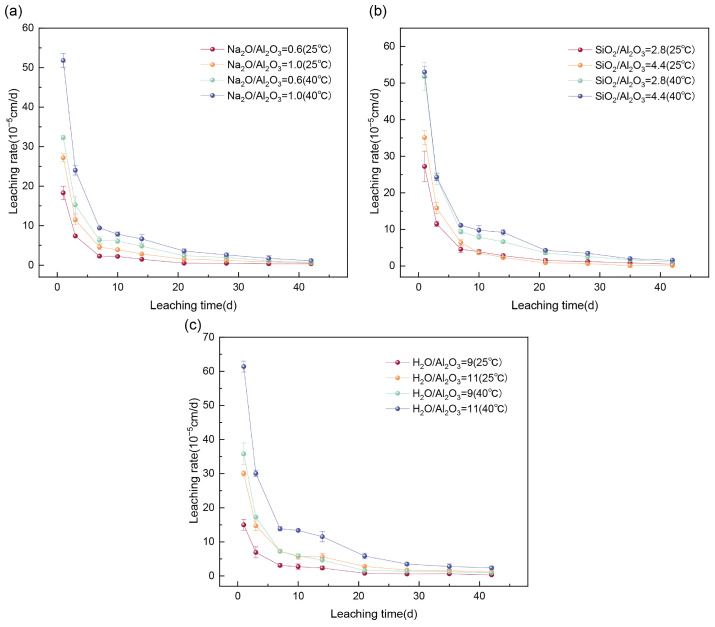
Leaching rate at different (**a**) Na_2_O/Al_2_O_3_, (**b**) SiO_2_/Al_2_O_3_, and (**c**) H_2_O/Na_2_O ratios at 25 °C and 40 °C.

**Figure 3:**
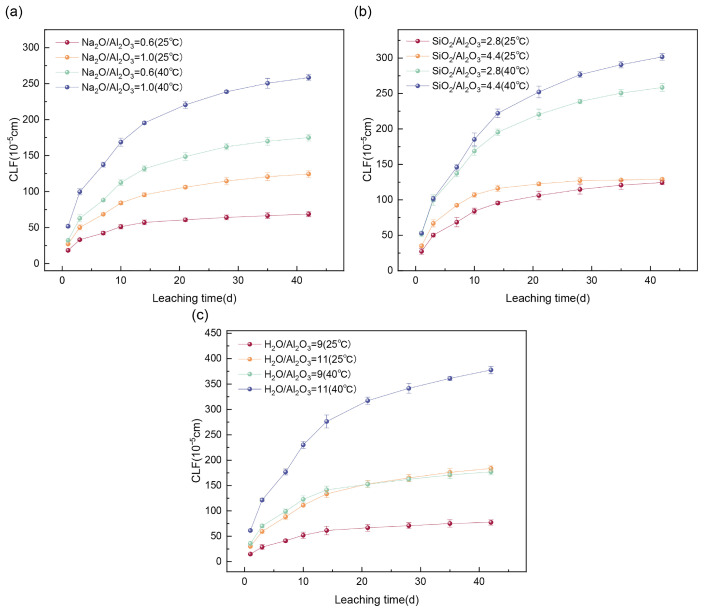
Cumulative leaching fraction (CLF) at different (**a**) Na_2_O/Al_2_O_3_, (**b**) SiO_2_/Al_2_O_3_, and (**c**) H_2_O/Na_2_O ratios at 25 °C and 40 °C.

The influence of Na_2_O/Al_2_O_3_ on the Cs^+^ leaching rate and cumulative leaching fraction is shown in Figure 2a and Figure 3a. Leaching experiments were conducted at 25 °C and 40 °C using 300 mL of deionized water as the leachant to investigate the effect of varying Na_2_O/Al_2_O_3_ ratios (0.6 and 1.0) on the Cs^+^ release from GP solidified forms. When the Na_2_O/Al_2_O_3_ ratio increased from 0.6 to 1.0, the 42-day cumulative leaching fraction of Cs^+^ increased by 80.79% at 25 °C and by 47.70% at 40 °C. This trend is primarily attributed to the reduced quantity of aluminate tetrahedra ([AlO_4_]) at higher Na_2_O/Al_2_O_3_ ratios, which decreases the density of Al–O–Si cross-linking sites. As a result, the network structure transitions from a rigid, highly cross-linked framework to a looser, chain-like configuration. This structural change increases porosity, enhances ion migration pathways, and ultimately elevates Cs^+^ leaching [34].

#### 3.1.2. Effect of SiO_2_/Al_2_O_3_ on Cesium Leaching

The effect of varying SiO_2_/Al_2_O_3_ ratios on the leaching resistance of the solidified forms is shown in Figure 2b and Figure 3b. Leaching tests were conducted at 25 °C and 40 °C using 300 mL of deionized water. When the SiO_2_/Al_2_O_3_ ratio was increased from 2.8 to 4.4, the 42-day cumulative leaching fraction of Cs^+^ showed a slight increase: 3.79% at 25 °C and 16.79% at 40 °C. This is due to the greater presence of Si–O–Si bonds at higher Si/Al ratios, which are more susceptible to hydrolysis in aqueous or acidic environments. This hydrolytic instability contributes to structural degradation and promotes cesium ion release [35].

#### 3.1.3. Effect of H_2_O/Na_2_O on Cesium Leaching

The effect of different H_2_O/Na_2_O ratios (9 and 11) on Cs^+^ leaching is illustrated in Figure 2c and Figure 3c. Leaching tests were performed at 25 °C and 40 °C using 300 mL of deionized water. Increasing the H_2_O/Na_2_O ratio from 9 to 11 led to a substantial rise in the 42-day cumulative leaching fraction of Cs^+^, with increases of 150.81% at 25 °C and 125.15% at 40 °C. Two main factors contribute to this observation: (1) a higher H_2_O/Na_2_O ratio increases the liquid-to-solid ratio, enhancing mass transfer and leaching efficiency; and (2) a higher water content results in greater evaporation of free water during curing, leading to increased capillary porosity and the formation of mesopores and macropores within the gel matrix. These larger pores facilitate ion migration and thus increase Cs^+^ leaching [36].

**Figure 4:**
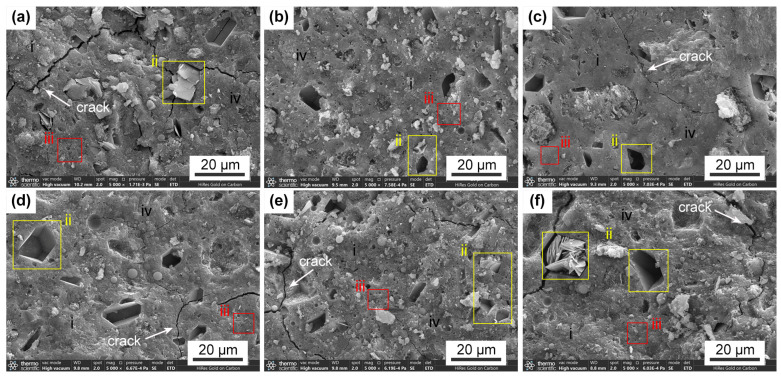
SEM images of geopolymer samples (scale: 20 μm): (**a**) S1; (**b**) S2; (**c**) S4; (**d**) S8; (**e**) S10; and (**f**) S12.

Across all of the single-factor experiments, the cumulative leaching fraction of Cs^+^ increased with temperature. At 40 °C, the leaching rate and cumulative leaching fraction for a given mix design were more than 1.5 times higher than those observed at 25 °C. Nevertheless, all of the measured values remained below the limits defined by Chinese national standards, indicating that the solidified blocks exhibited strong leaching resistance and the effective immobilization of Cs^+^. As shown in Figure 3, the cumulative leaching fraction increased rapidly during the first 27 days and then gradually stabilized. This behavior is attributed to the dominance of surface dissolution in the early stages, during which surface-bound Cs^+^ was preferentially released, as well as the ‘first flush’ phenomenon in cementitious systems that creates an immediate concentration gradient driving the initial rapid release of surface-bound species. Although longer-term data are needed to project the performance over geological timescales, the leaching process became primarily controlled by internal diffusion after 27 days. This diffusion proceeds at a significantly slower rate than surface dissolution, thereby reducing the leaching rate over time [37]. This behavior is a characteristic feature of well-structured geopolymer matrices. The low permeability and dense microstructure observed in the SEM analysis (Figure 4) support the potential for this diffusion-controlled regime to maintain low leaching rates over extended periods. This conclusion is strongly supported by Jain et al. [14], who’s study of fly ash-based geopolymers demonstrated that a diffusion-dominated mechanism effectively maintains low cesium leachability, with its performance improving over 180 days of curing.

### 3.2. Response Surface Analysis

#### 3.2.1. Analysis of Response Surface Models

The compressive strength and leaching test results for the fifteen experimental samples are presented in Table 3. The data were analyzed using Design–Expert 13 software, with nonlinear fitting performed using various models. Based on a comparison of the sum of squared deviations, fitting errors, and statistical metrics, the quadratic polynomial model was determined to provide the best fit [38]. This model effectively captures the interaction effects among variables and was therefore selected for further analysis in this study. The mathematical models corresponding to the 28-day compressive strength (Y_1_), the 42-day cumulative leaching fraction at 25 °C (Y_2_), and at 40 °C (Y_3_) are given in Equations (4)–(6), respectively. The statistical significance of these models was evaluated through the analysis of variance (ANOVA) test, and the results are summarized in Table 4.

Y_1_ = 17.55 + 2.54x_1_ − 3.30x_2_ + 6.67x_3_ − 1.04x_1_x_2_ + 0.88x_1_x_3_ − 0.48x_2_x_3_ + 0.08x_12_ − 0.81x_22_ + 0.51x_32_ (4)

Y_2_ = 137.12 + 21.40x_1_ + 15.02x_2_ + 41.20x_3_ + 10.10x_1_x_2_ + 16.87x_1_x_3_ + 13.25x_2_x_3_ + 10.32x_12_ − 55.57x_22_ + 33.90x_32_ (5)

Y_3_ = 255.30 + 28.71x_1_ + 32.54x_2_ + 88.84x_3_ + 44.72x_1_x_2_ + 12.07x_1_x_3_ + 27.50x_2_x_3_ + 32.88x_12_ − 94.41x_22_ + 80.44x_32_ (6)

**Figure 5:**
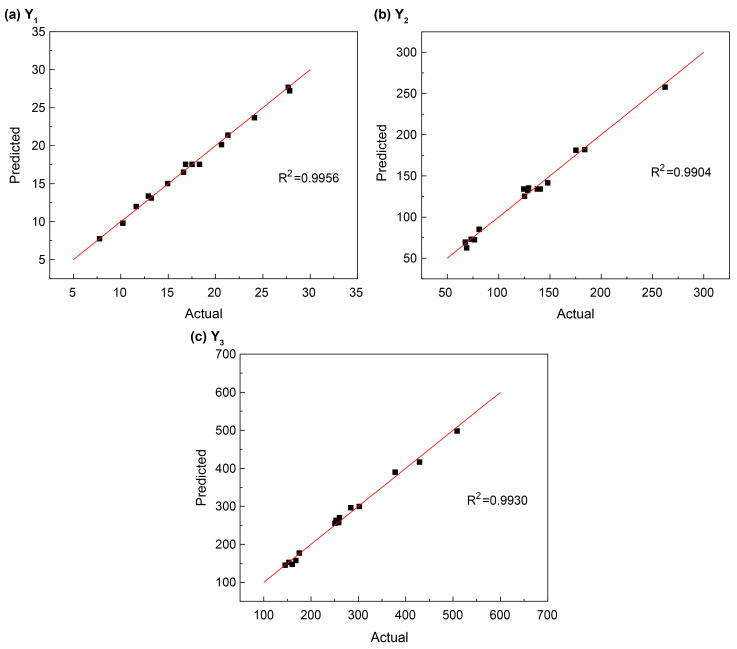
Comparison of experimental and predicted values of (**a**) 28-day compressive strength, (**b**) the 42-day cumulative leaching fraction at 25 °C, and (**c**) the 42-day cumulative leaching fraction at 40 °C.

A scatter plot was constructed with the experimental (actual) values on the horizontal axis and the predicted values on the vertical axis, as shown in Figure 5. Each point lies close to the line (y = x), indicating that the predicted values are in strong agreement with the measured values, and the model demonstrates a high degree of fit.

The model’s *p*-values (Prob > F) and F-values derived from the ANOVA are critical for evaluating the reliability of the selected models. A *p*-value < 0.05 indicates statistical significance, while *p* < 0.01 denotes high significance. In this study, the *p*-values for the Y_1_, Y_2_, and Y_3_ models were all <0.01 (Table 4), indicating highly significant regression models [39,40]. The coefficient of determination (R^2^) was used to verify the reliability of the polynomial equations. The R^2^ values for Y_1_, Y_2_, and Y_3_ were 0.9956, 0.9904, and 0.9930, respectively, meaning that only 0.44%, 0.96%, and 0.70% of the variability could not be explained by the models. Since all of the values are well below 10%, the simulation analysis based on these models is considered robust and feasible [41]. The lack-of-fit *p*-values for Y_1_, Y_2_, and Y_3_ were 0.6309, 0.5199, and 0.0530, respectively. As all of the *p*-values exceeded 0.05, the lack-of-fit tests confirmed that the models are reliable predictors of compressive strength and leaching behavior. Table 4 further shows that the three factors significantly influenced both the compressive strength and cumulative leaching fraction, with their interaction terms also playing measurable roles. The significance ranking of the factors affecting 28-day compressive strength was x_3_ > x_1_ > x_2_ > x_1_x_2_ > x_1_x_3_ > x_2_^2^ > x_3_^2^ > x_2_x_3_ > x_1_^2^.

For the 42-day cumulative leaching fraction at 25 °C, x_3_ > x_2_^2^ > x_3_^2^ > x_1_ > x_2_ > x_1_x_3_ > x_2_x_3_ > x_1_x_2_ > x_1_^2^. For the 42-day cumulative leaching fraction at 40 °C, x_3_ > x_2_^2^ > x_3_^2^ > x_2_ > x_1_x_2_ > x_1_ > x_1_^2^ > x_2_x_3_ > x_1_x_3_. H_2_O/Na_2_O (x_3_) and Na_2_O/Al_2_O_3_ (x_1_) had the most pronounced impact on compressive strength, likely due to the influence of water content on workability and pore structure. Although water does not directly participate in the reaction, it affects the binder distribution and microstructure formation. SiO_2_/Al_2_O_3_ (x_2_) was the second most influential factor; lower SiO_2_/Al_2_O_3_ ratios generally corresponded to higher compressive strengths. For leaching resistance, x_3_, x_2_^2^, and x_3_^2^ were the most significant terms, as reflected by their *p*-values (<0.0001 for x_3_ and x_2_^2^ in Y_2_, and <0.0001 for all three in Y_3_), which were well below the 0.05 threshold.

#### 3.2.2. Response Surface Plots

**Figure 6:**
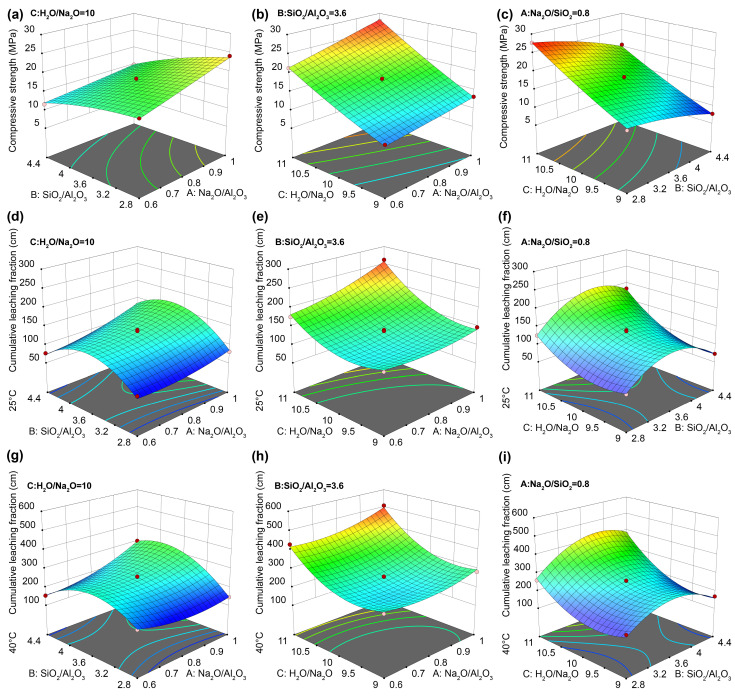
Response surface model of strength and cumulative fraction leached of geopolymer: (**a**,**d**,**g**) the relationship between Na_2_O/Al_2_O_3_ and SiO_2_/Al_2_O_3_; (**b**,**e**,**h**) the relationship between H_2_O/Na_2_O and Na_2_O/Al_2_O_3_; (**c**,**f**,**i**) the relationship between SiO_2_/Al_2_O_3_ and H_2_O/Na_2_O.

Response surface plots visually illustrate the interaction between two factors and their combined effect on the response variable. A greater surface curvature indicates a more significant interaction effect, whereas a flatter surface indicates weaker interaction [25]. The fitted response surfaces for the three key factors, namely, Na_2_O/Al_2_O_3_, SiO_2_/Al_2_O_3_, and H_2_O/Na_2_O, are shown in Figure 6.

Figure 6a displays the effect of the interaction between Na_2_O/Al_2_O_3_ and SiO_2_/Al_2_O_3_ on the 28-day compressive strength. When SiO_2_/Al_2_O_3_ is held constant, the compressive strength increases with rising Na_2_O/Al_2_O_3_. Conversely, when Na_2_O/Al_2_O_3_ is fixed, the compressive strength decreases with increasing SiO_2_/Al_2_O_3_. The response surface shows slight inclination and relative smoothness. The physical and mechanical properties of GPs are closely related to the SiO_2_/Al_2_O_3_ and Na_2_O/Al_2_O_3_ ratios [42]. The porosity of amorphous GPs, which directly controls mechanical strength, is strongly influenced by the Si/Al ratio. Within a certain range, GPs synthesized at lower SiO_2_/Al_2_O_3_ ratios exhibit lower porosity, a higher degree of polymerization, and greater structural stability than those synthesized at higher ratios [43], consistent with the observed response surface trend. However, excessively high Si/Al ratios in alkaline environments can hinder the dissolution and release of silica and alumina tetrahedral precursors, leaving unreacted metakaolin particles that agglomerate, thereby reducing the final strength of the GP [44].

Figure 6b illustrates the effect of the interaction between Na_2_O/Al_2_O_3_ and H_2_O/Na_2_O on the 28-day compressive strength. When Na_2_O/Al_2_O_3_ is held constant, the compressive strength increases approximately linearly with rising H_2_O/Na_2_O. The response surface is steep, with the maximum strength increasing by 256.70%. When H_2_O/Na_2_O is fixed, the strength increases with increasing Na_2_O/Al_2_O_3_, although the effect is less pronounced. These results indicate that H_2_O/Na_2_O is the dominant factor influencing the compressive strength of the solidified form. Within a certain range, increasing the solid-to-liquid ratio enhances the compressive strength. However, when this ratio exceeds an optimal threshold, the strength decreases sharply [45]. This decline is attributed to the reduced water content caused by the addition of KCuFC-Cs, which may result in the inadequate wetting of particles, negatively affecting the paste’s workability and formability. Furthermore, increasing SiO_2_/Al_2_O_3_ lowers the water content in the formulation, whereas increasing Na_2_O/Al_2_O_3_ raises it [36]. At high Na_2_O/Al_2_O_3_ ratios, the abundance of sodium ions creates a strongly alkaline environment, which can enhance polymerization by accelerating the dissolution of metakaolin and promoting gel formation [46].

Figure 6c shows the interaction between SiO_2_/Al_2_O_3_ and H_2_O/Na_2_O on 28-day compressive strength. When SiO_2_/Al_2_O_3_ is held constant, the compressive strength increases with rising H_2_O/Na_2_O, with a maximum improvement of 172.84%. Conversely, when H_2_O/Na_2_O is fixed, the compressive strength declines with increasing SiO_2_/Al_2_O_3_. The slope of the surface contours indicates that H_2_O/Na_2_O exerts a slightly greater influence than SiO_2_/Al_2_O_3_, consistent with the ANOVA results in Table 4.

Figure 6d,g depict the interaction between Na_2_O/Al_2_O_3_ and SiO_2_/Al_2_O_3_ on the 42-day cumulative leaching fraction. When SiO_2_/Al_2_O_3_ is constant, the cumulative leaching fraction increases slightly as Na_2_O/Al_2_O_3_ increases. When Na_2_O/Al_2_O_3_ is fixed, the cumulative leaching fraction initially increases and then slightly decreases with increasing SiO_2_/Al_2_O_3_. The curvature in different directions is negative and positive, forming a saddle-shaped response surface. This trend is due to variations in the pore structures formed by different SiO_2_/Al_2_O_3_ ratios. A higher ratio can hinder solubility and gel formation, resulting in unreacted metakaolin. Within the range of Si/Al = 3–4, a mesoporous structure is likely to form due to limited soluble silicates in the activating solution, as shown in Figure 4c–f [25], thereby increasing the cumulative leaching fraction. Additionally, increasing Na_2_O/Al_2_O_3_ may reduce the leaching resistance due to shrinkage caused by higher alkali content [25].

Figure 6e,h show the interaction between Na_2_O/Al_2_O_3_ and H_2_O/Na_2_O on the 42-day cumulative leaching fraction. When H_2_O/Na_2_O is held constant, the cumulative leaching fraction initially decreases slightly, then increases with rising Na_2_O/Al_2_O_3_. Instead, a saddle trend is observed: when Na_2_O/Al_2_O_3_ is fixed, the cumulative leaching fraction initially decreases slightly and then increases with increasing H_2_O/Na_2_O, mirroring the reverse pattern when H_2_O/Na_2_O is fixed and Na_2_O/Al_2_O_3_ varies. The steeper contour indicates that H_2_O/Na_2_O has a more substantial impact. The cumulative leaching fractions at 25 °C and 40 °C increased by 155% and 180%, respectively, at higher H_2_O/Na_2_O ratios. This behavior is attributed to the role of water in determining the structural integrity of the GP. Although only a small amount of water remains as interstitial water within the GP network, excessive water content leads to the evaporation of free water during curing, forming large pores and voids [36]. These voids, observed in Figure 4, reduce the leaching resistance of the solidified forms.

Figure 6f,i display the interaction between SiO_2_/Al_2_O_3_ and H_2_O/Na_2_O on the 42-day cumulative leaching fraction. When SiO_2_/Al_2_O_3_ is fixed, the cumulative leaching fraction first slightly decreases and then increases with the rising H_2_O/Na_2_O. When H_2_O/Na_2_O is fixed, the cumulative leaching fraction shows a similar trend with increasing SiO_2_/Al_2_O_3_. The response surface exhibits a saddle shape with a central depression, suggesting that both excessively high and low values of SiO_2_/Al_2_O_3_ and H_2_O/Na_2_O are detrimental to improving leaching resistance.

### 3.3. Optimization

#### Macro Performance

The multi-objective optimization problem, also known as the multi-criteria optimization problem, involves a set of objective functions and corresponding constraint conditions [47]. Based on the models established using RSM, the optimization was designed to maximize the 28-day compressive strength of the GP solidified form and minimize the 42-day cumulative leaching fractions at 25 °C and 40 °C. These three responses (Y_1_, Y_2_, and Y_3_) were treated as equally important. A numerical optimization technique based on the desirability function (d_j_) was employed to optimize each individual response. The desirability function (d_j_) was defined on a scale from zero to one, where zero indicates an unacceptable response and one corresponds to the target or most desirable response value. The overall desirability function was computed as the geometric mean of the individual desirability values. Multi-objective optimization thus corresponds to maximizing the overall desirability (D). The optimal mix ratios obtained were Na_2_O/Al_2_O_3_ = 0.84, SiO_2_/Al_2_O_3_ = 2.8, and H_2_O/Na_2_O = 10.26, with a desirability value of 0.901, indicating the strong reliability of the predicted results [48]. Experimental validation was carried out using these optimal ratios, with three replicate tests performed to calculate the mean, in accordance with GB/T 17671-2021 [31]. The mean of the three results was determined, and any result deviating by more than ±10% from the mean was to be discarded; however, no outliers were identified. The reported data represent the mean of the retained results, based on three replicates. As shown in Table 5, the deviations of each replicate from the mean for Y_1_, Y_2_, and Y_3_ were 4.70%, 5.87%, and 9.77%, respectively. All deviations were within the 10% tolerance specified by the Chinese National Standard (GB/T 17671-2021).

### 3.4. Microstructural Properties

**Figure 7:**
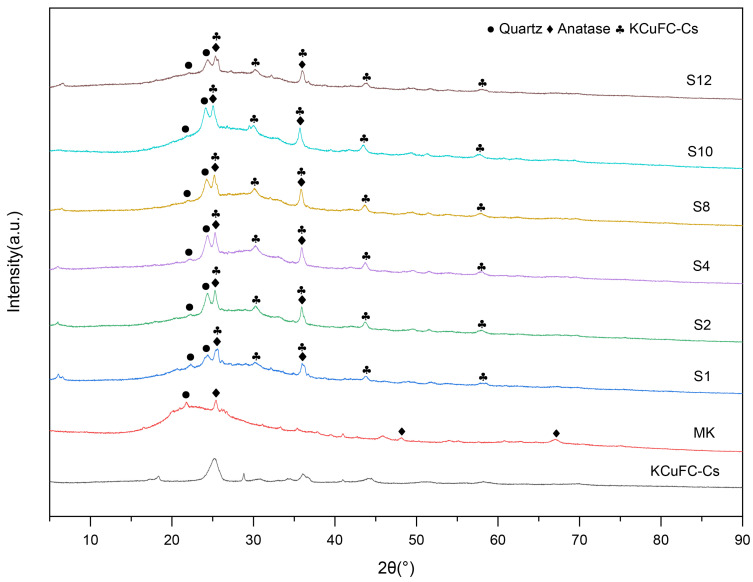
XRD patterns of metakaolin and geopolymer samples.

Figure 7 shows that the diffraction peak positions and intensities of the six sample groups did not vary significantly, indicating that the solidified forms exhibit similar phase compositions. The raw metakaolin contains crystalline phases such as quartz and anatase, and these phases are also present in the solidified products. In the XRD pattern of metakaolin, a broad diffraction peak within the range of 18° to 25° was related to the amorphous nature of metakaolin was identified. Following alkali activation, this amorphous signature evolved through measurable peak position shifts, confirming the transformation into a geopolymeric gel network [49]. The XRD patterns of the six GP groups (S1–S12) show that quartz peaks persist because of quartz’s low solubility in alkaline conditions, alongside other residual phases from the metakaolin precursor such as anatase, as confirmed by their standard reference patterns (ICPDS), including quartz (20.8° and 26.6° 2θ; PDF# 46-1045, ICPDS) and anatase (25.3°, 37.8°, 48.0°, and 68.8° 2θ; PDF# 21-1272, ICPDS). During geopolymerization, the amorphous structure of metakaolin is transformed from a layered silicate into a three-dimensional aluminosilicate gel network. The emergence of a characteristic amorphous hump is a hallmark of GP formation. The comparative analysis of samples S1 and S2 (increasing Na_2_O/Al_2_O_3_), as well as S10 and S12 (increasing H_2_O/Na_2_O), reveals that the 2θ values of the amorphous hump increase in both cases, suggesting a higher degree of geopolymerization [50]. This implies that the amorphous gel phase plays a dominant role in influencing the mechanical properties of alkali-activated metakaolin-based GPs.

Figure 4 displays the microstructure of the metakaolin-based GP solidified forms. All the investigated samples show similar structural characteristics, with the presence of gel-like solids indicating a typical GP microstructure. The geopolymers in this study are multi-component materials mainly composed of the following: (1) gel material (areas i and iv in Figure 4; (2) KCuFC-Cs and the voids resulting from the detachment of its particles (area ii in Figure 4); and (3) pores within the GP matrix (based on XRD analysis and morphology) (areas iii in Figure 4). Based on pore structure characterization via the BET method (see the Supplementary Materials), which provides quantitative data on specific surface area and pore volume distribution, along with SEM morphological analysis, the following microstructural features were observed across the samples. According to the pore parameters summarized in Supplementary Table S3, Sample S10 (Figure 4e) has the highest porosity, while Sample S8 (Figure 6d) has the lowest and the most complete GP structure, indicating the highest degree of geopolymerization. In S12 (Figure 4f), the increase in H_2_O/Na_2_O appears to promote more continuous gel formation, in agreement with the 28-day compressive strength response surface results. In samples S1 through S4 (Figure 4a–c), numerous pores are present on the surface. As both Na_2_O/Al_2_O_3_ and SiO_2_/Al_2_O_3_ increase, the porosity of the GP decreases and the overall density of the GP increases. This densification occurs because the alkaline dissolution of silicate and aluminate oligomers during geopolymerization enhances the polymerization reaction (Equation (4)). The resulting Si–O–Al covalent framework leads to the development of an amorphous to semi-crystalline GP structure. Thus, increasing the silicate content promotes a higher degree of geopolymerization and structural uniformity [51]. As a result, the proportion of homogeneous regions in the cross-section increases, and the matrix becomes denser, consistent with the XRD results.

However, in addition to small capillary pores, larger voids also influence the leaching performance. A comparison of samples S10 and S12 indicates that the microstructure of S12 (Figure 4f) appears more porous and permeable than that of S10 (Figure 4e), which may affect its cumulative leaching behavior. Additionally, the internal cracks observed in the SEM images originate from material-intrinsic factors, including drying shrinkage and chemical contraction during geopolymerization influenced by H_2_O/Na_2_O ratio variations, along with sample preparation effects such as stress propagation from cutting in the brittle matrix. These features, while noted, do not compromise the core findings on Cs^+^ immobilization.

(7) $$SiO_{4}^{4-}+Al{\left(OH\right)}_{4}^{-}\to {\left[Si-O-Al\right]}_{n}+OH^{-}+H_{2}O$$

**Figure 8:**
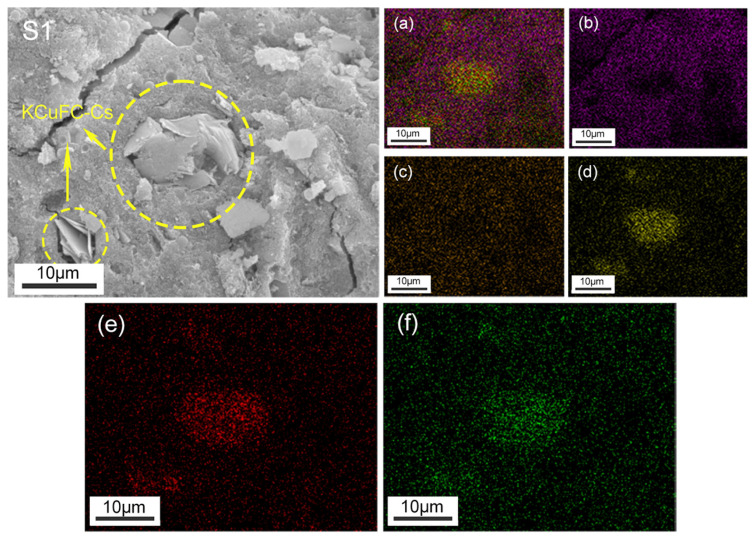
Energy dispersive X-ray spectroscopy (EDS) image and elemental distribution maps of S1: (**a**) EDS element mapping of S1; (**b**) Na; (**c**) K; (**d**) Cs; (**e**) Fe; (**f**) Cu.

Figure 8 presents the elemental distribution maps obtained from the EDS analysis of the internal cross-section of sample S1. The microstructural analysis suggests that effective Cs^+^ immobilization in metakaolin-based geopolymers (GPs) arises from a synergistic interplay of multiple mechanisms. The images reveal that Cs ions are primarily concentrated within the KCuFC adsorbent particles. However, a small amount of Cs^+^ is also uniformly dispersed throughout the surrounding GP matrix. The KCuFC-Cs particles appear to be well-encapsulated by the GP, which supports the effective immobilization of Cs^+^ within the solidified structure [43]. Beyond this physical entrapment, the ionic compatibility between Cs^+^ and alkali cations (Na^+^ and K^+^) in GP pores may facilitate ion exchange or electrostatic attraction, further enhancing retention [52]. XRD analysis confirms that immobilization occurs predominantly within the amorphous aluminosilicate network rather than through precipitation, as no new crystalline Cs-containing phases are detected, aligning with studies attributing high Cs selectivity in GPs to fixation within the cage-like sites of the aluminosilicate structure [53]. Collectively, these observations confirm that metakaolin-based GPs can securely fix Cs^+^ through the integration of physical encapsulation, chemical interaction, and structural confinement, effectively limiting its migration potential. This observation further confirms that metakaolin-based GPs can securely fix Cs^+^ and limit its migration potential.

**Figure 9:**
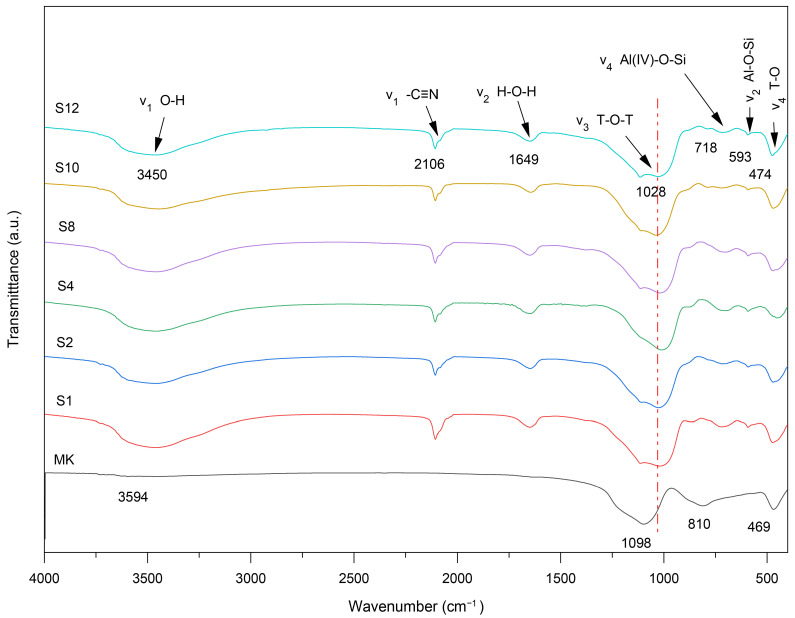
Fourier transform infrared spectroscopy spectra of metakaolin and geopolymer samples.

Figure 9 displays the FT-IR spectra of raw metakaolin and six groups of GP solidified forms. The broad absorption band between 3500 and 3000 cm^−1^ and the peak near 1650–1620 cm^−1^ are assigned to the stretching and in-plane bending vibration modes of water molecules, respectively. The band in the range of 1100–1000 cm^−1^ corresponds to the asymmetric stretching vibration of T–O–T bonds (where T represents Si or Al) [54]. In raw metakaolin, a strong peak at ~1100 cm^−1^, associated with Si–O asymmetric stretching, shifts to ~1010 cm^−1^ after geopolymerization. This downshift reflects a change in the local chemical environment of Si–O bonds and the formation of Al–O–Si linkages, suggesting that Al transitions from AlO_6_ octahedral coordination in metakaolin to AlO_4_ tetrahedral coordination in the GP network [55]. Additionally, a peak around 550–600 cm^−1^ is attributed to the symmetric stretching vibration of Al–O–Si, while the band near 450–500 cm^−1^ is associated with internal bending vibrations of T–O bonds [25]. A weak peak in the 2100–2200 cm^−1^ region is likely due to the stretching vibration of cyano groups (–C≡N) present in ferrocyanide. The details are provided in Table S2.

In metakaolin, the strong band at approximately 810 cm^−1^ corresponds to the stretching vibrations of hexa-coordinated Al(VI)–OH and Al(VI)–O bonds. This band nearly disappears after geopolymerization, and a new peak appears at ~710 cm^−1^, which is attributed to the bending vibration of tetra-coordinated Al(IV)–O–Si in cyclic structures. This shift confirms the conversion from Al(VI) to Al(IV) coordination, characteristic of the formation of the geopolymeric aluminosilicate framework [56]. The structural integrity of the GP is governed primarily by ionic and covalent bonding. As the Si/Al ratio increases, the number of Si–O–Si bonds grows, improving mechanical strength more effectively than Si–O–Al or Al–O–Al linkages [57]. At higher SiO_2_/Al_2_O_3_ ratios, the GP matrix becomes enriched with polysilicate siloxane and disiloxane structures (Si–O–Si), enhancing network connectivity. Notably, samples S2 and S8 exhibit the most significant peak shifts and intensities, indicating a higher degree of geopolymerization. The main spectral bands also shift toward higher wavenumbers, consistent with the XRD and response surface results.

## 4. Conclusions

### 4.1. Summary and Findings

This study investigated the optimal combination of key mix parameters for the GP-based solidification of KCuFC after adsorption of simulated Cs^+^ nuclide. Using RSM, the effects of Na_2_O/Al_2_O_3_, SiO_2_/Al_2_O_3_, and H_2_O/Na_2_O on the 28-day compressive strength and 42-day cumulative leaching fractions at 25 °C and 40 °C were systematically analyzed. A multi-objective optimization model was developed to enhance the overall performance of the solidified form. Additionally, single-factor experiments and microstructural analyses provided insights into the influence of each parameter on the internal structure and mechanical properties of the GP matrix. The main conclusions are as follows:

(1) A quadratic polynomial regression model was established to describe the relationships between mix parameters and the compressive strength and leaching behavior of the solidified forms. The model exhibited a high degree of fit, as demonstrated by the strong agreement between the predicted and experimental results, validating its applicability for mix proportion design.

(2) The experimental results revealed that H_2_O/Na_2_O ratio was the most significant factor affecting both the strength and leaching resistance, followed by the Na_2_O/Al_2_O_3_ ratio and SiO_2_/Al_2_O_3_ ratio. Microstructural analyses (XRD, SEM-EDS, and FT-IR) confirmed that lower H_2_O/Na_2_O ratio reduced paste workability, leading to heterogeneous agglomeration and reduced specimen compactness. The Na_2_O/Al_2_O_3_ ratio influenced system alkalinity and positively affected polymer setting and hardening. In contrast, excessive SiO_2_/Al_2_O_3_ ratio inhibited dissolution of silica and alumina precursors, thereby hindering matrix densification.

(3) Multi-objective optimization using RSM identified the optimal mix design as Na_2_O/Al_2_O_3_ = 0.84, SiO_2_/Al_2_O_3_ = 2.8, and H_2_O/Na_2_O = 10.23. Under these conditions, the experimental compressive strength was 20.95 MPa, and the 42-day cumulative leaching fractions at 25 °C and 40 °C were 7.906 × 10^−4^ cm and 1.5923 × 10^−3^ cm, respectively. The percentage errors between the predicted and experimental values were all below 10%, confirming the reliability and robustness of the optimized model.

In summary, while previous studies have focused primarily on immobilizing spent adsorbents, the relationship between GP mix design and synergistic solidification performance remains underexplored. This study provides a potential solution for immobilizing Cs-containing adsorbents and offers a quantitative approach for optimizing solidification performance. It also establishes the material’s fundamental immobilization capability under high-concentration conditions, providing crucial baseline data for subsequent applied research with actual waste samples. Moreover, given that intermediate- and high-level radioactive wastewater often contains multiple radionuclides, this methodology may serve as a valuable reference for the immobilization of other species, such as Sr^2+^ and Co^2+^.

### 4.2. Outlook and Future Work

This study successfully demonstrates the optimization of a metakaolin-based GP for the short-term solidification of Cs-laden KCuFC. However, to fully assess its viability for real-world applications, two key areas must be further investigated:

(1) Long-term Stability: Future work will involve accelerated aging tests such as thermal cycling and exposure to more aggressive leachants, with the mechanical and leaching properties monitored over a period of 6 to 12 months. This approach will allow for the development of predictive models of the material’s performance over geological timescales.

(2) Immobilization Mechanisms: To elucidate the precise mechanisms at the atomic level, advanced characterization techniques such as solid-state nuclear magnetic resonance spectroscopy and X-ray photoelectron spectroscopy will be employed. These techniques can probe the chemical environment of Cs^+^ and identify its binding sites within the GP matrix.

## Figures and Tables

**Table 1:** Central composite design for three factors at three levels.

Levels	Coded Factors	Actual Factors		
		Na_2_O/Al_2_O_3_(A)	SiO_2_/Al_2_O_3_(B)	H_2_O/Na_2_O(C)
Low	−1	0.6	2.8	9
0	0	0.8	3.6	10
High	1	1	4.4	11

**Table 2:** Chemical composition of metakaolin and silica fume (wt%).

Composition	SiO_2_	Al_2_O_3_	Ti_2_O	Fe_2_O_3_	ZrO_2_	Na_2_O	CaO	K_2_O	P_2_O_5_	MgO
MK	45.07	52.06	1.25	0.42	0.03	0.24	0.08	0.20	0.25	0.40
Silica fume	97.38	0.23	0.04	0.43	1.26	-	0.08	0.04	0.39	0.15

**Table 3:** Response surface test results of metakaolin-based geopolymer.

Mixture Code	Type	Na_2_O/Al_2_O_3_	SiO_2_/Al_2_O_3_	H_2_O/Na_2_O	28-Day Compressive Strength (MPa)	42-Day Cumulative Fraction Leached (10^−5^ cm)	
						25 °C	40 °C
S1	A1B1C2	0.6	2.8	10	16.61	68.82	175.04
S2	A3B1C2	1	2.8	10	24.10	124.42	258.54
S3	A1B3C2	0.6	4.4	10	11.61	76.52	153.04
S4	A3B3C2	1	4.4	10	14.93	129.13	301.94
S5	A1B2C1	0.6	3.6	9	10.20	128.25	252.79
S6	A3B2C1	1	3.6	9	13.21	147.67	284.04
S7	A1B2C3	0.6	3.6	11	20.62	175.27	429.08
S8	A3B2C3	1	3.6	11	27.83	262.19	508.59
S9	A2B1C1	0.8	2.8	9	12.90	67.58	160.01
S10	A2B3C1	0.8	4.4	9	7.76	73.29	167.73
S11	A2B1C3	0.8	2.8	11	27.68	125.13	259.94
S12	A2B3C3	0.8	4.4	11	21.07	183.82	377.64
S13	A2B2C2	0.8	3.6	10	16.83	140.37	257.09
S14	A2B2C2	0.8	3.6	10	17.51	134.42	258.54
S15	A2B2C2	0.8	3.6	10	18.30	137.56	250.28

**Table 4:** Variance analysis (ANOVA) of experimental results: (y_1_) 28-day compressive strength; (y_2_) the 42-day cumulative leaching fraction at 25 °C; (y_3_) the 42-day cumulative leaching fraction at 40 °C.

Response	y_1_	y_2_	y_3_
Model			
Standard deviation	0.67	8.66	14.79
R^2^ value	0.9956	0.9904	0.9930
F value	126.22	57.22	79.02
*p*-value (Prob > F)	<0.0001	0.0002	<0.0001
Lack of fit (Prob > F)	0.6309	0.5199	0.0530
*p*-value (Prob > F)			
x_1_-Na_2_O/Al_2_O_3_	<0.0001	0.0009	0.0027
x_2_-SiO_2_/Al_2_O_3_	0.0001	0.0045	0.0016
x_3_-H_2_O/Na_2_O	<0.0001	<0.0001	<0.0001
x_1_x_2_	0.0262	0.0670	0.0018
x_1_x_3_	0.0462	0.0115	0.1637
x_2_x_3_	0.2101	0.0281	0.0137
x_12_	0.8262	0.0706	0.0079
x_22_	0.0661	<0.0001	<0.0001
x_32_	0.2037	0.0007	0.0001

**Table 5:** Experimental validation results of metakaolin-based geopolymer using the optimized mixture.

Response		Optimized Mixture (FSM)		
		Experimental Value	Predicted Value	Error (%)
28-Day Compressive strength (MPa)		23.41	22.36	4.70%
42-Day Cumulative Fraction Leached (10^−5^ cm)	25 °C	79.06	74.68	5.87%
	40 °C	159.23	145.06	9.77%

## Data Availability

The original contributions presented in this study are included in the article/Supplementary Material. Further inquiries can be directed to the corresponding author.

## Supplementary Materials

The following supporting information can be downloaded at https://www.mdpi.com/article/10.3390/ma19071469/s1. Table S1: Main chemical reagents used in the experiments; Table S2: FT-IR absorption bands and their corresponding functional group assignments for the transformation products; Table S3: Summary of N2-physisorption data for samples; Figure S1: (a) N2 adsorption–desorption isotherms, and (b) pore width distribution plot of S1, S2, S4, S8, S10, S12.

## Abbreviations

The following abbreviations are used in this manuscript:

GP	Geopolymer
RSM	Response Surface Methodology
PB	Prussian Blue
GPMs	Geopolymer Microspheres
CV	Coefficient of Variation
CLF	Cumulative Leaching Fraction
MK	Metakaolin
XRD	X-ray Diffraction
SEM	Scanning Electronic Microscopy
EDS	Energy Dispersive X-ray Spectroscopy
FTIR	Fourier Transform Infrared Spectroscopy
ICP-MS	Inductively Coupled Plasma Mass Spectrometry
