# Supplementary material for: Optimization Design of Metakaolin-Based Geopolymer Solidification for Potassium Copper Hexacyanoferrate After Cs+ Adsorption Using Response Surface Methodology
Source: Materials (Basel). 2026 Apr 7;19(7):1469. doi: 10.3390/ma19071469 (PMC13074866; doi:10.3390/ma19071469)
Supplement: Supplementary file 1 [file materials-19-01469-s001.zip › materials-4192812-supplementary.pdf]

Optimization Design of Metakaolin-Based Geopolymer Solidification for Potassium Copper Hexacyanoferrate After Cs^+ Adsorption Using Response Surface Methodology

Table S1 Main chemical reagents used in the experiments.

Experimental reagent	Specifications	Manufacturer
Cupric nitrate	AR	Maclean Biochemical Technology Co.
Potassium hexacyanoferrate	AR	Maclean Biochemical Technology Co.
Cesium chloride	AR	Maclean Biochemical Technology Co.
Metakaolin	-	Shanxi Chaopai Calcined Kaolin Co., Ltd.
NaOH	AR	Zhengzhou Hongteng Chemical Co., Ltd.
Sodium silicate solution	AR	Jiaxing Yurui Refractory Chemical Co., Ltd.
Silicon dioxide powder	98%SiO2	Henan Hengyuan New Material Technology Co., Ltd.

Table S2 FT-IR absorption bands and their corresponding functional group assignments for the transformation products.

Wavenumber(cm−1)	Functional group
3450	O-H(v_1)
2106	-C≡N(v_1)
1649	H-O-H(v_2)
1028	T-O-T (T=Si/T=Al) (v_3)
718	Al(IV)-O-Si(v_4)
593	Al - O - Si(v_2)
474	T-O (T=Si or T=Al) (v_4)

Table S3 Summary of N_2-physisorption data for samples.

Mixture code	BET specific surface area(m^2/g)	BJH average pore diameter(4V/A),nm
S1	24.25	11.49
S2	21.18	10.78
S4	36.72	10.76
S8	10.78	15.43
S10	46.34	7.37
S12	31.10	11.51

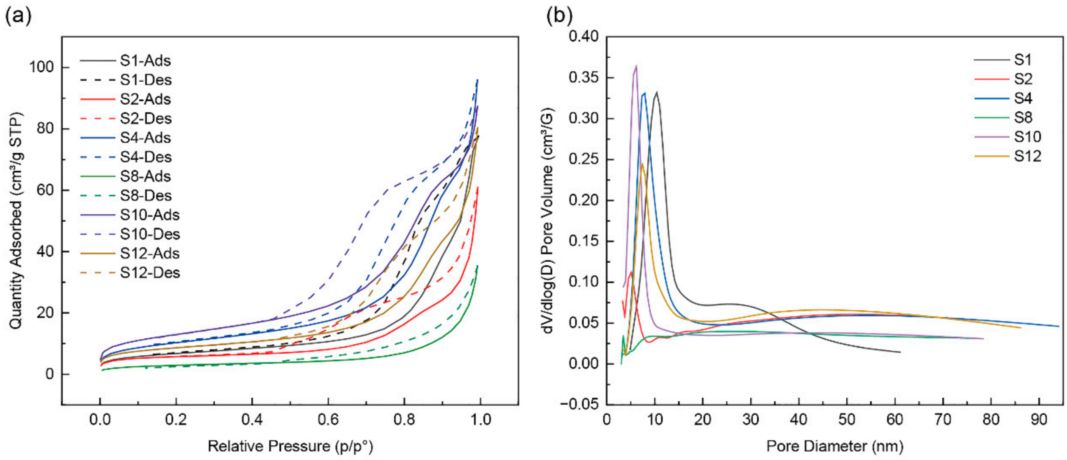

Figure. S1 (a) N_2 adsorption–desorption isotherms, and (b) pore width distribution plot of S1, S2, S4, S8, S10, S12.
